# New Perspective: Bench to Bedside Evidence of the Role of CD8+ T Cells in Alzheimer's Disease

**DOI:** 10.1002/iid3.70380

**Published:** 2026-03-06

**Authors:** Yong Peng, Shun‐yu Yao, Si‐Liang Wu, Huan Yang, Xiuli Zhang, Sugimoto Kazuo, Jia Liu, Miao‐qiao Du, Lan‐xin Lin, Xu‐hui Kang, Dai‐yi Jiang

**Affiliations:** ^1^ Department of Neurology First Affiliated Hospital of Hunan Traditional Chinese Medical College (Hunan Provincial Directly Affiliated Hospital of Traditional Chinese Medicine) Zhuzhou Hunan China; ^2^ Department of Geriatric Medicine First Affiliated Hospital of Hunan Traditional Chinese Medical College (Hunan Provincial Directly Affiliated Hospital of Traditional Chinese Medicine) Zhuzhou Hunan China; ^3^ Department of Neurology Provincial Hospital of Traditional Chinese Medicine Affiliated to Hunan University of Traditional Chinese Medicine Zhuzhou Hunan China; ^4^ Department of Neurology Xiangya Hospital, Central South University Changsha Hunan China; ^5^ Center for Science and Technology Innovation Hunan University of Chinese Medicine Changsha China; ^6^ Department of Neurology, Dongzhimen Hospital Beijing University of Chinese Medicine Beijing China; ^7^ Institute for Brain Disorders at Beijing University of Chinese Medicine Beijing China

**Keywords:** Alzheimer's disease, amyloid beta protein, animal models, autoantigens, CD8‐positive T‐lymphocytes, clinical trials, cognitive dysfunction, tau proteins

## Abstract

**Introduction:**

Amyloid beta plaques and tau tangles are the primary hallmarks of Alzheimer's disease (AD). Recently, passive anti‐Aβ immunotherapy for AD has markedly advanced, as supported by evidence from AD animal models and clinical trials. Whereas innate immunity significantly contributes to AD pathology, it does not fully represent the immune mechanisms linked to this condition. Therefore, focus should be directed toward adaptive immunity, encompassing both humoral and cellular immunity.

**Methods:**

Relevant publications and clinical trial data up to February 2026 were systematically reviewed to summarize the mechanisms, therapeutic targets, safety profiles, and translational applications of CD8+ T cells in AD.

**Results:**

Clinical and animal studies have particularly suggested a potential involvement of T cells in AD pathogenesis. T cells that infiltrate the central nervous system (CNS) exert both protective and detrimental effects on neural tissue in AD. Because autoreactive CD8+ T cells are generally expected to have cytotoxic effects on CNS cells, they have received less attention. Nevertheless, accumulating evidence suggests that CD8+ Treg cells are involved in various diseases.

**Conclusion:**

However, the function of anti‐Aβ‐specific CD8+ T cells in Alzheimer's disease (AD) remains ambiguous. Many subsets of CD8+ T cells have been well‐studied in autoimmunity. We suggest that CD8+ T cell subsets identified in AD studies may constitute a promising area for future AD research.

## Graphical abstract

Emerging evidence highlights a previously underappreciated role of CD8^+^ T cells in Alzheimer's disease (AD). Beyond amyloid‐β and tau pathology, CD8^+^ T‐cell subsets infiltrate the central nervous system and exhibit functional heterogeneity. Cytotoxic effector CD8^+^ T cells may exacerbate neuroinflammation and neuronal injury, whereas regulatory or exhausted phenotypes may exert immunomodulatory effects. Findings from transgenic mouse models and clinical immune profiling of peripheral blood and cerebrospinal fluid demonstrate clonal expansion and antigen experience of CD8^+^ T cells. These translational insights suggest that targeting CD8^+^ T‐cell responses could represent a complementary immunotherapeutic strategy in AD.

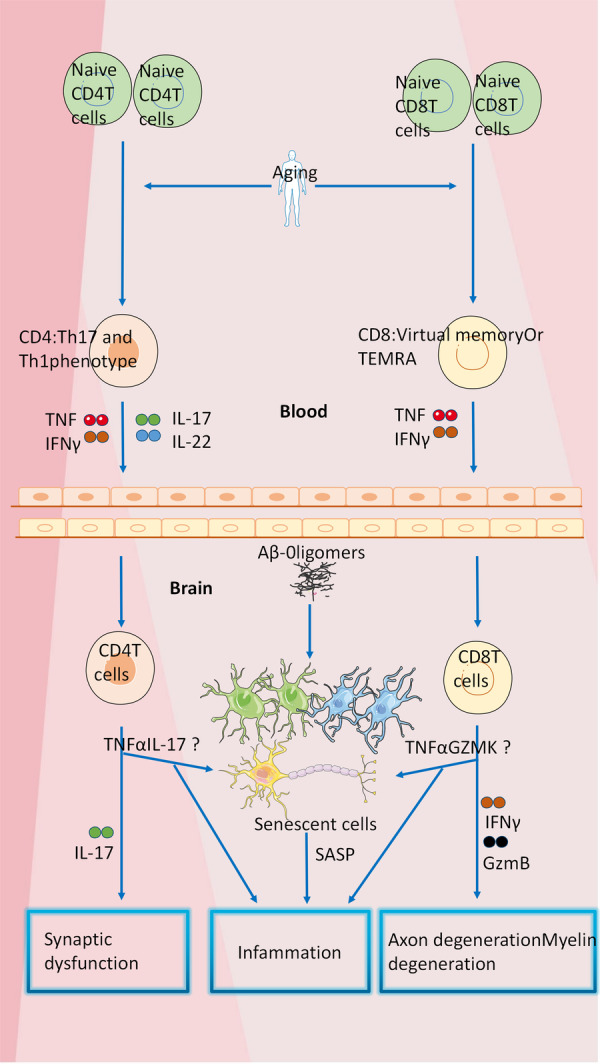

**Abbreviations**
Ach: acetylcholine
AChE: acetylcholinesterase
AChEI: acetylcholin‐esterase inhibitor
AD: Alzheimer's disease
AE: adverse effects
APP: amyloid precursor protein
ARIA: amyloid‐related imaging abnormalities
ARIA‐E: amyloid‐related imaging abnormalities ‐Edema
Aβ: beta amyloid protein
BACE‐1: β‐site amyloid precursor protein cleaving enzyme 1
BBB: blood‐brain barrier
FDA: Food and Drug Administration
GSA‐3β: glycogen synthase kinase3β
iGluRs: ionic receptors
MCI: mild cognitive impairment
ME: meningoencephalitis
mGluRs: metabolic receptors
MPT: mitochondrial permeability transition
NbM: basal ganglia of memory
NFTs: neuro‐cell tangles
NMDAR: anti‐N‐methyl‐d‐aspartate receptor
TEAEs: treatment‐related adverse reactions
The membrane‐associated C99: comprising the terminal 99 amino acid residues of the carbon chain"
VGLUT: vesicular glutamate transporter

## 1 Introduction

In Alzheimer's disease (AD), the presence of amyloid beta (Aβ) plaques and tau tangles [1, 2, 3] are major contributors to AD pathogenesis [4, 5, 6], with Aβ pathology representing the primary factor in the early stage [7, 8, 9. Specifically, AD is distinguished by the accumulation of the 40–42 amino acid peptide Aβ (Aβ40‐42) in amyloid plaques [10, 11, 12]. Thus, Aβ‐targeting immunotherapies are considered more important than those targeting tau [13, 14].

Recently, a marked progress has been made in passive anti‐Aβ immunotherapy for AD, supporting the role of immunological factors in AD pathology [1]. The antibodies used include lecanemab [Le qembi], donanemab [15, 16], and aducanumab [Aduhelm]) [17], among others [2]. However, in contrast to the current state of immunotherapies for neuro‐autoimmune diseases such as multiple sclerosis (MS), evidence supporting disease‐modifying and complete treatment effects of immunotherapies for AD remains lacking [7, 18, 19, 20].

Substantial evidence has supported the critical role of immunological factors in the pathology of AD, including innate immunity [1, 21, 22, 23], adaptive immunity [24], and particularly T cells, mostly including CD4+ and CD8+ T cells. Among these, regulatory T cells (Tregs) have a protective role [25, 26, 27, 28].

## 2 Brief Progress on Anti‐Aβ Immunotherapies

The role of anti‐Aβ immunotherapies has been extensively examined in AD animal models, including Aβ‐precursor protein (APP) transgenic mice [9, 29, 30, 31, 32], as well as in clinical trials [9, 33, 34, 35, 36], in which reduced cerebral Aβ content was confirmed by positron‐emission tomography (Aβ‐PET) [9, 15]. Moreover, anti‐Aβ immunotherapies decrease tau species and glial fibrillary acidic protein in the cerebrospinal fluid (CSF) or blood [15, 16, 37, 38, 39], reducing Aβ loading in the CSF and Aβ‐associated tauopathy and astrocytic activation in the brain [29].

## 3 Brief Progress on Immunity and AD

Given the clear importance of anti‐Aβ immunotherapies in AD, the relevant immune mechanisms should be clearly established. Essentially, immunity can be divided into innate immunity and adaptive immunity [1, 40, 41, 42, 43]. The local immune system, including the complement system, microglia, and astrocytes, played a role in these processes [23, 44, 45, 46], resulting in neuronal injury and death [47].

Collectively, although innate immunity plays a crucial role in AD pathology [48], it does not account for all immunological factors implicated in AD pathogenesis [49]. Therefore, the contribution of adaptive immunity, particularly the involvement of T cells, must also be examined to fully understand AD pathogenesis.

## 4 T Cells and AD

Adaptive immunity encompasses humoral immunity, mainly governed by B cells, and cellular immunity, influenced by T cells. Research suggests that T cells play a crucial role in the pathogenesis of AD [24]. Individuals with AD exhibit increased levels of CD4+ T helper (Th) cells, T regulatory cells (Tregs, particularly FoxP3+ CD4+ T cells), Th9 cells, and Th17 cells and decreased levels of CD8+ cytotoxic T cells in peripheral blood [50, 51, 52].

CNS‐inﬁltrating T cells may play a protective role for neurons in AD [24] by expressing neurotrophic factors, enhancing microglial phagocytic activity, and helping to reduce Aβ deposition [53]. However, some Aβ‐reactive T cells may also exacerbate AD progression through secretion of pro‐inﬂammatory cytokines, thus leading to sterile, chronic inﬂammation [53]. Moreover, in the APP/PS1 mouse model of AD, Th1‐derived interferon (IFN) γ was shown to impair cognitive function by promoting microglial stimulation and increasing Aβ aggregation. Treatment with an anti‐IFNγ antibody could alleviate disease progression in these mice, which supported the view that Th1 cells play a neurotoxic role in AD [54]. Furthermore, Aβ‐specific Th2 cells can inhibit cytokine production by glial cells, whereas Aβ‐specific Th1 cells exhibit the capability to stimulate the production of pro‐inflammatory cytokines [55]. Oxidative medicine and cellular longevity by microglial cells might also be an important factor contributing to AD pathology [53]. According to this body of evidence, we speculate that diﬀerent stages of AD progression may be characterized by distinct proﬁles of T cell subpopulations and by opposing roles of immune cells [24].

Tregs can inhibit the release of reactive oxygen species (ROS) from microglia and prevent ROS‐induced neuronal damage [56]. In individuals with AD, peripheral blood exhibits increased levels of CD4+ T helper (Th) cells [57], T regulatory cells (Tregs), Th9 cells, and Th17 cells, whereas the number of CD8+ cytotoxic T cells is diminished [57]. A recent phase I clinical trial with a small cohort of PD patients has initiated an exploration of sargramostim, a recombinant human granulocyte‐macrophage colony‐stimulating factor that augments Treg‐mediated suppression [58].

Aβ1‐42 peptide injection into rat brains led to increased levels of IL‐17A, RORγt, and IL‐22; this was reversed by the anti‐inflammatory action of TGF‐β1 [59], indicating that Th17 cells and their related cytokines act in concert to enhance neuroinflammation and degeneration in AD.

Conversely, a number of studies have demonstrated that the activation of T cells may diminish symptoms of AD. Anti‐PD‐1 therapy improved cognitive deficits and Aβ plaque loading in the hippocampus and cortex of an AD mouse model [60, 61] (Baruch, Deczkowska et al. 2016). Effector T cells may suppress AD pathology by stimulating Aβ clearance by macrophages and microglia [62].

**Figure 1:**
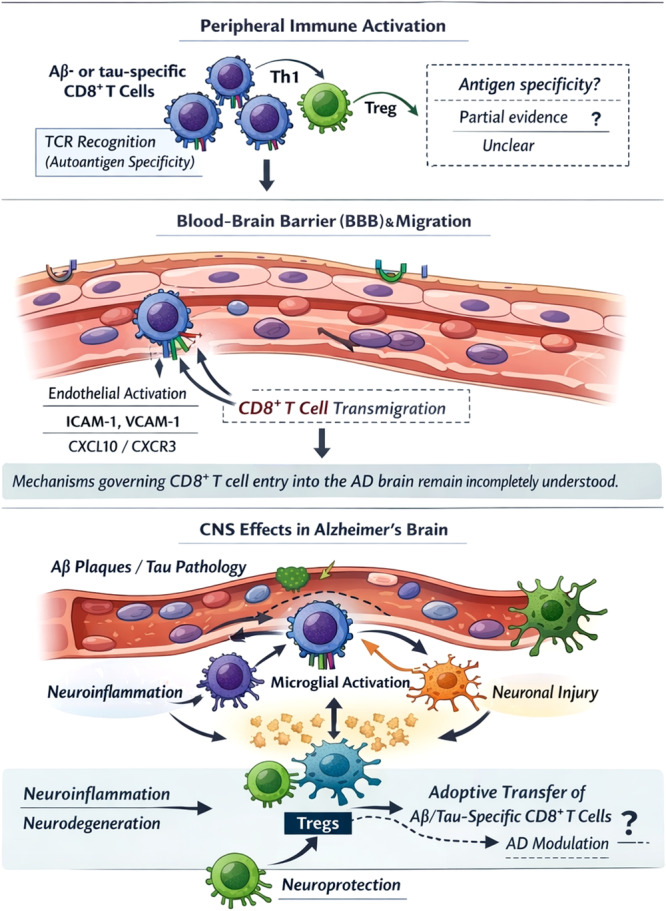
The relationship of T cells and the pathogenesis of AD. T cells could play and injury or protective role on neurons, depending on T cell subsets and their related cytokine, and local immune cells. AD, Alzheimer's disease.

From a classic immunological view, the following evidence should be presented to confirm the role of T cells, as we have previously reported for MG, MS, uveitis, and type 1 diabetes [63, 64, 65, 66, 67] (i) they should be autoantigen‐specific (Aβ‐ or tau‐speciﬁc), (ii) the profile of cytokines secreted by T cells should be present, and (iii) adoptive transfer should induce or inhibit the disease. These have been addressed by some reports [68, 69, 70]; however, more evidence generated following this protocol is required in the future to unravel the precise mechanisms associated with T cells in AD (Figure 1).

### 4.1 Aβ‐Speciﬁc T Cells in AD

To date, the role of adaptive immune T cells in the development of AD remains unclear [71]. In peripheral tissues, T cells are stimulated by modified self‐peptide fragments of Aβ and tau (such as Aβ1‐42), which are displayed by antigen‐presenting cells like microglia and dendritic cells located within the brain parenchyma (Chaffey 2003, 2020). In AD, effector T cells modified self‐peptide fragments of Aβ and tau and induced self‐clonal expansion [28, 72], supported through evidence from the periphery and brain in patients with AD [50, 52, 62, 73], as well as from studies of mixed microglia‐astroglia cultures [74] and APP/PS1 mice [71]. Interestingly, in APP/PS1 mice, Aβ‐reactive Teff cells showed stable Th1 and Th17 profiles and enhanced memory impairment and systemic inflammation, which could be downregulated by Aβ‐reactive Tregs derived from peripheral blood and located in the brain [71].

In Table 1, we have compiled recent findings on autoreactive T cells in AD, encompassing both animal models and clinical investigations. Unfortunately, most studies were focused on CD4+ T cells, whereas only a few were focused on CD8+ T cells.

**Table 1:** Studies of Aβ‐specific T cells in AD.

References	Cells	Auto‐antigen	Animal models or patients	Results
Machhi et al. [71]	Aβ reactive Th1 and Th17 cells (CD4^+^ cells)	Aβ 15–30 peptide	APP/PS1 mice	1. Stable and long‐lived Aβ specific‐Th1 and Aβ‐Th17 clones. 2. Adoptive transferred Aβ‐Th1 and Aβ‐Th17 clones into APP/PS1 mice could enhance memory impairment and systemic infammation, increase amyloid burden, elevate microglia activation, and exacerbate neuroinfammation. 3. Aβ specific‐Tregs in peripheral blood and CNS could inhibit memory impairment and systemic inflammation.
Yang et al. [75]	Aβ reactive CD4^+^ Tregs(CD4^+^ cells)	Aβ 1–42 peptide	3xTg‐AD mice	1. Aβ‐specific Tregs inhibited microglial proinflammatory activity and modulated the microglial phenotype via bystander suppression. 2. Single adoptive transfer of Aβ+ Tregs could inhibit cognitive impairments, Aβ accumulation, hyper‐phosphorylation of tau, and neuroinflammation during AD pathology. 3. Aβ‐specific Tregs effectively inhibited inflammation in primary microglia induced by Aβ exposure.
Yeapuri et al. [28]	TCR_Aβ_‐Tregs(CD4^+^ CD25^+^ cells)	Aβ 15–30 peptide	APP/PS1 mice	1. TCR_Aβ_‐Tregs expressed an Aβ‐specifc TCR. 2. Adoptive transfer of TCR_Aβ_‐Tregs led to sustained immune suppression, reduced microglial reaction, and amyloid loads. 3. ^18^F‐fuorodeoxyglucose radiolabeled TCR_Aβ_‐Treg homed to the brain facilitating antigen specifcity. Reduction in amyloid load was associated with improved cognitive functions
Fisher et al. [76]	Aβ reactive Th1 cells (CD4^+^ cells)	Aβ 1–42 peptide	APP/PS1 mice, APPSwe/PS1dE9 Tg mice	1. Th1, Th2, and Th17 CD4 T cells were injected ICV to migrate from the lateral ventricles into the brain parenchyma in mice. It was shown that primarily Th1 cells cross the ependymal layer of the ventricle and migrate within the brain parenchyma by stimulating an IFN‐γ–dependent dialogue with neural cells, which maintains the effector function of the T cells. 2. When injected into a mouse model of Alzheimer's disease, Aβ‐specific Th1 cells target Aβ plaques, increase Aβ uptake, and promote neurogenesis with no evidence of pathogenic autoimmunity or neuronal loss.
Rosset et al. [77]	Aβ‐specific Tc1 cells (CD8+ T cell)	Aβ 33–41 peptide	APP/PS1 mice, B6 wild‐type mice	1. Aβ33‐41 was naturally processed and presented in association with H‐2‐Db molecule on neurons and CD11b+ microglia. 2. Upon optimization of anchor residues for enhanced binding to H‐2‐Db, immunization with the modified Aβ33‐41NP peptide elicited Aβ‐specific IFNγ‐secreting CD8+ T cells, which are cytotoxic towards Aβ‐expressing targets. 3. Whereas T cell infiltration in the brain of APPPS1 mice is dominated by CD3+ CD8− T cells and increases with disease evolution between 4 and 7 months of age, a predominance of CD3+ CD8+ over CD3+ CD8− cells was observed in 6‐ to 7‐month‐old APPPS1 but not in WT animals, only after vaccination with Aβ33‐41NP. The number of CD11b+ mononuclear phagocytes, which significantly increases with age in the brain of APPPS1 mice, was reduced following immunization with Aβ33‐41NP. Despite peripheral activation of Aβ‐specific CD8+ cytotoxic effectors and enhanced infiltration of CD8+ T cells in the brain of Aβ33‐41NP‐immunized APPPS1 mice, no clinical signs of severe autoimmune neuroinflammation were observed.
Eremenko et al. [78]	Aβ reactive Th1 cells (CD4^+^ cells)	Aβ 1–42 peptide	5XFAD Tg mice, B6 wild‐type mice	1. The BDNF‐secreting Aβ‐T cells migrated efficiently to amyloid plaques, where they significantly in creased the levels of BDNF, its receptor TrkB, and various synaptic proteins known to be reduced in AD. 2. The injected mice demonstrated reduced levels of beta‐secretase 1 (BACE1)—a protease essential in the cleavageprocessoftheamyloidprecursorprotein—andamelioratedamyloidpathologyandinflammationwithin the brain parenchyma
Fisher et al. [79]	Aβ reactive Th1 cells (CD4^+^ cells)	Aβ 1–42 peptide	Aβ PP‐Tg mice, AβPP/IFN‐ B6SJLF1 Tg mice. C57BL6 and SJL mice	1. Following Aβ immunization, CD11c^+^ dendritic cells (DCs) and CD4^+^ T cells occurred primarily in the perivascular and leptomeningial spaces of cerebral vessels deposited with Aβ. 2. CD11c+ cells expressed high levels of the DCmaturation markers DEC‐205, MHC class II and CD86. Notably, the majority of cerebral blood vessels were found adjacent to Aβ plaques, expressing high levels of the ICAM‐1 and VCAM‐1 adhesion molecules. 3. The drainage of Aβ to the leptomeningeal and perivascular spaces and its deposition there provide the antigenic source for DCs to stimulate Aβ ‐specific T cells on their way to target amyloid plaques within the brain tissue.
Monsonego et al. [80]	Aβ reactive Th1 cells (CD4^+^ cells)	Aβ 1–40 and Aβ 1–42 synthetic peptides	B6 wild‐type mice	4. IFN‐γ‐treated microglia serve as efficient Aβ APCs of both Aβ 1‐40 and Aβ 1‐42, mediating CD86‐dependent proliferation of Aβ ‐reactive T cells. 5. Aβ‐reactive Th1, but not Th2, cells, underwent apoptosis after stimulation, which was accompanied by increased levels of FN‐γ, NO, and caspase‐3. T cell apoptosis was prevented in the presence of an inducible NO synthase type 2 inhibitor [81]. 6. Microglia‐mediated proliferation of Aβ‐reactive Th2 cells was associated with expression of the Th2 cytokines IL‐4 and IL‐10, which counterbalanced the toxic levels of NO induced by A. Our results demonstrate NO‐dependent apoptosis of T cells by Aβ ‐stimulated microglia which may enhance CNS innate immune responses and neurotoxicity in AD. Secretion of NO by stimulated microglia may underlie a more general pathway of T cell death in the CNS seen in neurodegenerative diseases. 7. Furthermore, Th2 type T cell responses may have a beneficial effect on this process by down‐regulation of NO and the proinflammatory environment.
Zhao et al. [82]	Aβ reactive Th1 cells (Total T cells	Aβ 1–40 and Aβ 1–42 peptides	3xTg‐AD, APP/PS1, C57BL/6 mice	1. TECs not only play a critical role in supporting T cell development but also mediate the deletion of autoreactive T cells by expressing autoantigens. 2. ESCs can be selectively induced to differentiate into TEPs in vitro that further develop into TECs *in vivo* to support T cell development. 3. Transplantation of mouse ESC (mESC)‐TEPs into AD mice reduced cerebral Aβ plaque load and improved cognitive performance, in correlation with an increased number of T cells, enhanced CP gateway activity, and increased number of macrophages in the brain. 4. Transplantation of the APP −/− mESC‐TEPs results in a more effective reduction of AD pathology as compared to wild‐type (APP+/+) mESC‐TEPs. This is associated with the generation of Aβ‐specific T cells, which leads to an increase of anti‐Aβ antibody (Ab)‐producing B cells in the spleen and enhanced levels of anti‐Aβ Ab in the serum, as well as an increase of Aβ phagocytosing macrophages in the CNS. They suggest that transplantation of APP−/− human ESC‐ or induced pluripotent stem cell (iPSC)‐derived TEPs may provide a new tool to mitigate AD in patients.
Park et al. [83]	Aβ reactive Tregs (CD4^+^ CD25^+^ cells)	Aβ 1–42 peptide	5xFAD mice	1. CD4+CD25+ Tregs, isolated after Aβ stimulation and expanded using a G‐rex plate with a gas‐permeable membrane, were adoptively transferred into 5xFAD mice. 2. levels of Aβ, phosphorylated tau (pTAU), and nitric oxide synthase 2 (NOS2) in the hippocampus. Real‐time RT‐PCR was employed to assess the mRNA levels of pro‐ and anti‐inflammatory markers. 3. Aβ‐specific Tregs not only improved cognitive function but also reduced Aβ and pTAU accumulation in the hippocampus of 5xFAD mice. They also inhibited microglial neuroinflammation. These effects were observed at doses as low as 1.5 *×* 103 cells/head.
Saresella et al. [84]	PD1‐expressing Aβ reactive CD4^+^ T cells,	Aβ peptides: 1–40, 1–6, and 1–35	AD and MCI patients HC	1. PD1‐expressing CD4 T cells, density of PD‐L1 on CD14 APC, IL‐10 production, and PD‐L1‐expressing/IL‐10‐producing CD14 APC were significantly reduced in AD and MCI patients compared to HC. Aβ ‐stimulated PD1/AV‐expressing (apoptotic) CD4 T cells were also diminished,. 2. Proliferation was augmented in AD and MCI patients compared to controls. Finally, incubation of cells with PD‐L1‐neutralizing Ab significantly decreased apoptosis of Aβ‐specific CD4 T cells. 3. An impairment of the PD‐L1/PD1 pathway is present in AD and MCI. Such alteration results in reduced IL‐10 production and diminished apoptosis of A ‐specific CD4 T cells.
Ethell et al. [85]	Aβ reactive T cells (Total T cells,	Aβ 1–42 peptide	F3 APP/PS1 mice,	1. A restricted Aβ‐specific immune re‐activation can provide cognitive and pathological benefits to APPsw + PS1 transgenic mice for at least 2.5 months. 2. A single infusion of Aβ‐specific immune cells from Aβ vaccinated littermates improved performance in cognitively impaired APP + PS1 mice. Recipients had lower levels of soluble Aβ in thehippocampus, less plaque‐associated microglia, and more intense synaptophysin immunoreactivity, compared with untreated controls. 3. However, Aβ‐specific infusates enriched for Th1 or depleted of CD4+ T‐cells were not effective, nor were ovalbumin‐specific infusates. These benefits occurred without global or brain‐specific inflammatory responses. Chronically high levels of AB can cause immune tolerance, hypo‐responsiveness, or anergy to Aβ, but our findings demonstrate that Aβ‐specific immune cells can resume endogenous Aβ‐lowering processes and may be an effective Aβ therapeutic.
Gate et al. [62]	CD3+CD8+CD45RA+T cells	Aβ1–40 and Aβ1–42 peptides	AD and MCI patients HC	1. peripheral blood mononuclear cells and discovered an immune signature of AD that consists of increased numbers of CD8+TEMRA cells. 2. In a second cohort, CD8+ TEMRA cells were negatively associated with cognition. Furthermore, single‐cell RNA sequencing revealed that TCR signalling was enhanced in these cells. 3. By using several strategies of single‐cell TCR sequencing in a third cohort, clonally expanded CD8+ TEMRA cells in the cerebrospinal fluid of patients with AD. Finally, we used machine learning, cloning and peptide screenst demonstrate the specificity of clonally expanded TCRs in the CSF of AD patients to two separate EBV antigens. These results reveal an adaptive immune response in the blood and CSF in AD and provide evidence of clonal, antigen‐experienced T cells patrolling the intrathecal space of brains affected by age‐related neurodegeneration [86].

*Note:* Aβ: amyloid beta, Th1: type 1 T helper, Th17: type 17 T helper, APP/PS1: mouse/human amyloid precursor protein and mutant human presenilin 1, Tregs: regulatory T cells, Teff: effector T cells, CNS: central nervous system. 3xTg‐AD mice: B6;129‐Tg (APPSwe, tauP301L)1Lfa *Psen1tm1Mpm*/Mmjax) mice, TCR_Aβ_‐Tregs: Tregs expressing a transgenic TCRAβ, BDNF: brain‐derived neuro trophic factor, Aβ PP‐Tg mice: amyloid‐ protein precursor (AβPP)‐transgenic (Tg) mice, AβPP/IFN‐ B6SJLF1 Tg mice: Homozygous IFN‐Tg mice: crossed with AβPP‐Tg mice, TECs: Thymic epithelial cells, ESCs: embryonic stem cells, TEPs: thymic epithelial progenitors, CP: choroid plexus, APP: amyloid precursor protein. APP −/−: APP gene deleted, Ab: antibody, MCI: mild cognitive impairment, HC: healthy controls, F3 APP/PS1 mice:a cross between either heterozygous APPsw mice and heterozygous PS1mice or from a cross between APPsw + PS1 mice and non‐transgenic mice, TEMRA: T effector memory CD45RA+, TCR: T cell receptor, CSF: cerebrospinal fluid, EBV: Epstein–Barr virus. Aβ refers to amyloid beta; Th1 denotes type 1 T helper cells; Th17 indicates type 17 T helper cells; APP/PS1 signifies mouse and human amyloid precursor protein along with mutant human presenilin 1; Tregs represents regulatory T cells; Teff stands for effector T cells; and CNS corresponds to the central nervous system.3xTg‐AD mice: B6;129‐Tg (APPSwe, tauP301L)1Lfa Psen1tm1Mpm/Mmjax mice, TCRAβ‐Tregs: Tregs with a transgenic TCRAβ, BDNF: brain‐derived neurotrophic factor, AbPP‐Tg mice: amyloid‐beta precursor protein (AbPP)‐transgenic mice, AbPP/IFN‐B6SJLF1 Tg mice: Homozygous IFN‐transgenic mice crossed with AbPP‐Tg mice, TECs: thymic epithelial cells, ESCs: embryonic stem cells, TEPs: thymic epithelial progenitor cells, CP: choroid plexus, APP: amyloid precursor protein.“APP −/−: APP gene knockout, Ab: immunoglobulin, MCI: mild cognitive decline, HC: healthy subjects, F3 APP/PS1 mice: offspring from either heterozygous APPsw and heterozygous PS1 mice or from APPsw + PS1 mice crossed with non‐transgenic animals, TEMRA: T effector memory cells CD45RA+, TCR: T cell antigen receptor, CSF: cerebrospinal fluid, EBV: Epstein‐Barr virus.”

### 4.2 Aβ‐speciﬁc CD8+ T Cells in AD

The role of anti‐Aβ‐specific CD8+ T cells in AD remains unclear [87]. Nonetheless, CD8+ T cells remain significant; a clinical Aβ vaccine trial using AN1792 was unsuccessful owing to meningoencephalitis onset in several patients with AD, as verified by postmortem examination [88]. This adverse reaction may be associated with CD4+ and/or CD8+ T cells, alongside substantial macrophage infiltration and reduced amyloid deposition (Unger, Li et al. 2020, Piehl, van Olst et al. 2022) [68, 69, 89, 90].

To date, primary AD immunotherapy trials have utilized humanized anti‐Aβ monoclonal antibodies or vaccination strategies employing the N‐terminal region of Aβ to induce antibody production while circumventing Aβ‐specific T cell activation. Most AD immunotherapy trials have failed, suggesting that more attention should be given to the role of Aβ‐specific T cells in AD. Anti‐Aβ mAbs such as bapineuzumab [91, 92] and solanezumab [93] were ineffective in enhancing cognitive function in patients with AD.

The epitope of Aβ‐specific CD4+ T cells is known in patients with AD [94] and animal models. Epitopes derived from Aβ that stimulate CD4+ T cell responses have also been identified in various mouse genotypes and HLA class II transgenic mice [95, 96, 97, 98].

Immunization with the full Aβ1‐42 peptide could potentially activate both Aβ‐specific CD4+ and CD8+ T cells. Given that autoreactive CD8+ T cells are anticipated to be cytotoxic toward central nervous system cells, they have been less emphasized in AD research. Nonetheless, CD8+ Tregs have been demonstrated to be involved in various diseases [99, 100, 101], including our previous study [102].

The CD8+ T cell epitope for the Aβ1‐42 peptide remains controversial. One study has reported the Aβ15‐30 peptide (KLVFFAEDVGSNKGA) in mice, as well as the Aβ15‐42 amino acid region in humans [103]. Another study in B6 mice suggested the Aβ33‐42 peptide. Nonetheless, an additional study in BALB/c mice demonstrated that Aβ12‐28 serves as the CD8+ T cell epitope. Consequently, the precise CD8+ T cell epitope of the Aβ1‐42 peptide remains undetermined. Different epitopes of the Aβ1‐42 peptide may be targeted in different mouse strains, such as Aβ1‐28 in BALB/c mice and Aβ1‐28 or 17‐40 in DRB0101 mice, which are transgenic for human MHC Class II molecules [97]. Please see Table 2.

**Table 2:** Evidences of CD8 T cells in AD.

Type	Reference	CD8 T cell source	Animal model or AD subtype	CD8 T subset	Major result	Institution
Clinical studies	[62]	PBMCs, CSF	97 healthy individuals, 31 patients with MCI, 28 patients with AD and 8 patients with PD	CD8 + T cell, TEM and TCM CD8 + T cells, CD8 + TEMRA cells	1. Performed mass cytometry of PBMCs and discovered an immune signature of AD that consists of increased numbers of CD8 + TEMRA cells. 2. CD8 + TEMRA cells were negatively associated with cognition. 3. Single‐cell RNA sequencing revealed that TCR signalling was enhanced in these cells. 4. Clonally expanded CD8 + TEMRA cells in the CSF of AD patients. 5. By machine learning, cloning and peptide screens to demonstrate the specificity of clonally expanded TCRs in the CSF of AD patients to two separate EBV antigens.	Stanford University, University of California at San Francisco, University of California at San Diego, California, USA
	Lu et al. 2021	PBMCs, AD brain tissues	ImmPort database, A bulk sequencing dataset of AD brain tissues	CD8 + T cell,	1. Eight clusters were identified, including memory CD4 T, NKT, NK, B, DC, CD8 Tcells, and platelets. 2. NK cells were significantly decreased in patients with AD, while CD4 T cells were increased. 3. NK and DC cells exhibited the highest IRG activity. GO and KEGG analyses of the scRNA and bulk sequencing data showed that the DEGs focused on the immune response. 4. Seventy common IRGs were found in both peripheral NK cells and the brain. 5. Seventeen TFs were associated with IRG expression, and the PPI network indicated that STAT3, IRF1, and REL were the hub TFs.	Tongji Medical College, Huazhong University of Science and Technology, Wuhan, China,
	[50]	PBMCs	Healthy young and healthy elderly individuals, AD patient	CD8 + T cell,	1. The distribution of peripheral T cell subsets in young and healthy old people is markedly different, characterized by decreased numbers of naive cells and increased numbers and clonal expansions of memory cells, predominantly in the CD8 + MHC class I‐restricted subset. 2. Dramatic alterations in naive and memory subsets of CD4+ cells in patients with mild AD, with greatly decreased percentages of naiıve cells, elevated memory cells, and increased proportions of CD4+ but not CD8+ cells lacking the important costimulatory receptor CD28. CD4+CD25high potentially T regulatory cells with a naive phenotype are also reduced in AD patients.	University of Tubingen, T ¨ ubingen, Germany; Medical University of Gdansk, Gda ´ nsk, Poland; Sir Mortimer B. Davis Jewish General Hospital, Montreal, QC, Canada;; University of Sherbrooke, Sherbrooke, QC, Canada
	[104]	Infiltrating T cells	Single‐nuclear RNA sequencing data of the middle temporal gyrus from 84 donors with AD (*n* = 42) or healthy control (n = 42) w	CD8 + T cell,	1. Using recent large‐scale, high‐quality single‐nuclear sequencing datasets from over 84 AD and control cases, single‐nuclear RNAseq data from 800 lymphocyt were collected from 70 individuals to complete unbiased molecular profiling. 2. effector memory CD8 T cells are the major lymphocyte subclass enriched in the brain tissues of individuals with AD dementia. 3. disease‐enriched interactions involving CD8 T cells and multiple brain cell subclasses including two distinct microglial disease states that correlate, respectively, to beta‐amyloid and tau pathology. 4. beta‐amyloid‐associated microglia are a major hub of multicellular cross‐talk gained in disease, including interactions involving both vulnerable neuronal subtypes and CD8 T cells. 5. Amyloid‐response microglia are depleted in APOE4 carriers. Overall, these human‐based studies provide additional support for the potential relevance of effector memory CD8 T cells as a lymphocyte population of interest in AD dementia and provide new candidate interacting partners and drug targets for further functional study.	University of California, Los Angeles, Los Angeles, CA, USA;
	[105]	Single‐cell ATAC‐seq RNA‐seq data	29 AD patients and 26 age and sex‐matched Healthy controls	CD8 + T cell,	1. Using single cell sequencing strategies, including assay for transposase‐accessible chromatin and RNA sequencing. it was shown that a striking amount of open chromatin in peripheral immune cells in AD. In CD8 T cells, and also shown a cis‐regulatory DNA element co‐accessible with the CXC motif chemokine receptor 3 gene promoter. 2. a novel AD‐specific RELA transcription factor binding site is adjacented to an open chromatin region in the nuclear factor kappa B subunit 2 gene. 3. apolipoprotein E genotype‐dependent epigenetic changes in monocytes. 4. differentially accessible chromatin regions in genes associated with sporadic AD risk.	Northwestern University Feinberg School of Medicine, Chicago, IL, USA
	[69]	CSF	45 healthy controls,14 patients with MCI or AD	CD8 T effector memory cells	1. single cell RNA sequencing on CSF from 45 cognitively normal subjects ranging from 54–82 years old, it was shown that upregulation of lipid transport genes in monocytes with age. 2. compared this cohort to 14 cognitively impaired subjects, downregulation of lipid transport genes in monocytes occurred concomitantly with altered cytokine signaling to CD8 T cells in cognitively impaired subjects. 3. Clonal CD8 T effector memory cells upregulated CXCR6 in cognitively impaired subjects. The CXCR6 ligand, CXCL16, was elevated in CSF of cognitively impaired subjects,	Northwestern University Feinberg School of Medicine, Chicago, IL, USA; Stanford University School of Medicine, Stanford, California, USA; Icahn School of Medicine at Mount Sinai; University of California at San Diego, La Jolla, CA, USA.
	[106]	PBMCs, CSF	8 AD patients and 4 age and sex‐matched Healthy controls	CD69 + CD103 + CD8 + T cells,	The frequency of CD69+CD103+CD8 + T cells was strikingly higher in the CSF than in the peripheral blood (among memory fraction, 13.5% vs 0.11%, difference (mean [SE]): 13.4% [2.9]). This CD69+CD103+CD8 + T‐cell population was increased in the CSF from patients with chronic inflammatory diseases, including multiple sclerosis and with neurodegenerative diseases such as Parkinson disease and Alzheimer's disease compared with controls (11.5%, 13.0%, 8.1% vs 2.9%, respectively). By contrast, the frequency was not altered in acute inflammatory conditions in the CNS (4.0%). Single‐cell RNAseq analysis confirmed Trm signature in CD69+CD103+CD8 + T cells in the CSF, supporting their Trm‐like phenotype, which was not clear in controls.	National Institute of Neuroscience, National Center of Neurology and Psychiatry, Kodaira, Japan; Kyoto University; Kansai Medical University Medical Center, Moriguchi, Japan
Animal Studies	Wang et al. 2024		C57BL/6 J mice, APP/PS1 mice (B6. Cg‐Tg(APPswe, PSEN1Δ9) 85Dbo/J), 5xFAD mice (B6. CgTg; APPSwFILon, PSEN1* M146L* L286V), congenic JHT mice (B6.129P2‐Igh‐Jtm1Cgn/J), and 5xFAD‐BKO mice	CXCR6 + CD39 + CD73 + / − CD8 + TRM‐like cells	1. By artificially blocking or augmenting CD8 + T cells in the brain of 5xFAD mice, it was shown that AD‐like pathology is promoted by pathogenic, proinflammatory cytokines and exhaustion markers expressing CXCR6 + CD39 + CD73 + /−CD8 + TRM‐like cells. 2. The CD8 + T cells appear to act by targeting disease associated microglia (DAM), and CD8 + T cells were shown that in tight complexes with microglia around Aβ plaques in the brain of mice and humans with AD. 3. These CD8 + T cells were induced by B cells in the periphery, further underscoring the pathogenic importance of the adaptive immunity in AD. 4. CD8 + T cells and B cells should be considered as therapeutic targets for control of AD, as their ablation at the onset of AD is sufficient to decrease CD8 + T cells in the brain and block the amyloidosis‐linked neurodegeneration	Laboratory of Molecular Biology and Immunolgy, Baltimore, MD; National Institute on Aging, Baltimore, MD; University of Massachusetts Medical School, Worcester, MA; The Leslie and Susan Gonda Multidisciplinary Brain Research Center, Israel; The Mina and Everard Goodman faculty of Life sciences, Israel; Bar‐Ilan University, Ramat Gan, Israel; University of Pennsylvania, Philadelphia, PA; Ludwig‐Maximilians‐Universität München, Munich, Germany; LMU Munich, Munich, Germany
	[107]	PBMCs, Infiltrating T cells	5×FAD mice	Infiltrating CD8 + T cells	1. Using single‐cell RNA‐sequencing, it showed that infiltration of T cells into AD cultures led to induction of IFN = γand neuroinflammatory pathways in glial cells. 2. CXCL10 and its receptor, CXCR3 played a key role in regulating T cell infiltration and neuronal damage in AD cultures	Massachusetts General Hospital, Harvard Medical School, Shriners Burns Hospital, Boston, MA, USA
	[108]	PBMCs, Infiltrating T cells	SAMP8 mice	Infiltrating CD8 + T cells	1. With a dose‐dependent profile, salidroside ly attenuated cognitive impairment, reduced the accumulation of Aβ plaques and restored neuronal damage. 2. Salidroside also suppressed the infiltration of CD8 + T cells, oxidative stress, and inflammatory cytokines, and improved mitochondrial metabolism, iron metabolism, lipid metabolism, and redox in the SAMP8 mice brain.	Zhu Jiang Hospital, Southern Medical University, Guangzhou, China; China Academy of Chinese Medical Sciences, Beijing, China
	Zeng et al. 2025	Database reanalysis	N/A	CD8 T cells	1. ATP6V1D, ATP6V1G2, CLTB, and NSF were identified as biomarkers, exhibiting a positive correlation with each other and a downregulated expression in AD. 2. activated CD8 T cells and various dendritic cells (DCs) is associated with an inflammatory milieu in AD while also displaying a negative correlation with the biomarkers.	Shanxi Medical University, Taiyuan, Shanxi, China
	Kang et al. 2025	Brain parenchymal, choroid plexus	5xFAD mice	CD8+ resident memory T cells (TRM)	1.11,587 single cells and found distinct differences in T cell and choroid plexus cell populations between 5xFAD mouse and littermate control were analyzed. 2. Subsequent sub‐clustering of T cells in the 5xFAD mouse revealed distinct subtypes, with CD8+ resident memory T cells (TRM) being the most prevalent T cell type. 3. an increase in T cell exhaustion markers, including Pdcd1, Ctla4, and Havcr2, with a particularly significant elevation of PD‐1 and TIM‐3 in CD8 + TRM in 5xFAD mouse. 4. Choroid plexus (ChP) epithelial cells showed altered gene expression patterns, with higher expression of MHC class I and Type I IFN‐stimulated genes in 5xFAD mouse compared to the control mouse, suggesting an association with clonal expansion of AD‐specific T cells in the brain. 5. Through single‐cell RNA sequencing (scRNA‐seq) analysis, it suggested that the potential role of resident memory CD8 + T cell and their possible interactions with ChP epithelial cells.	Seoul National University Graduate School, Seoul, Republic of Korea; PB Immune Therapeutics Inc., Seoul, Republic of Korea; Pusan National University, Yangsan, Republic of Korea
	[109]	Brain	Nur77GFP‐APP/PS1 mice, C57BL/6JTg, Tg(APPswe, PSEN1dE9) 85Dbo/Mmjax) mice, “wt” (Nur77GFP:C57BL/6‐Tg(Nr4a1‐EGFP/cre)820Khog/J mice).	CD8 T cells	1. Using flow cytometry to characterize T cell populations and their activation mode in an AD mouse model. 2. By assessing GFP expression in C57BL/6JTg(Nr4a1‐EGFP/cre)820Khog; Tg(APPswe, PSEN1dE9)85Dbo/Mmjax mice, antigenia depended from antigen‐independent activation in CD4⁺, CD8⁺, and double‐negative T cells (DNTs). 3. This approach allows analysis of the full repertoire of antigen‐specifically activated T cells in a physiological immune system without prior knowledge of target antigens. 4. AD‐like amyloid pathology progression was monitored by monthly scoring until mice reached 2, 6, 10‐12 or 15‐18 months of age and Aβ‐quantification via thioflavine S staining. Antigen‐specific activation during AD development was assessed by comparing AD mice with wildtype littermates. 5. At 15–18 months, AD mice exhibited elevated numbers of activated, highly differentiated DNTs, along with increased antigen‐specific CD8⁺ and DNT cells relative to controls.	University Medicine, Greifswald, Germany
	[110]	Brain	3xTg‐AD, and WT B6129SF2/J	brain CD8 + T cell, CD103–CD8 + T cells	1. The brain CD8 + T cell compartment is dysregulated in AD patients and in the 3xTgAD mouse model, accumulating activated CD103–tissue‐resident memory T cells that produce large amounts of GrK. 2. These CD103–CD8 + T cells originate from the circulation and migrate into the brain using LFA‐1 integrin. Ablation of brain CD103–CD8 + T cells in 3xTg‐AD mice ameliorates cognitive decline and reduces neuropathology. 3. GrK induces neuronal dysfunction and tau hyperphosphorylation in human and mouse cells via PAR‐1, which is expressed at higher levels in the AD brain, revealing a key immune‐mediated neurotoxic axis.	University of Verona, Verona, Italy
	Zhang et al. 2024	PBMCs	58 AD patients,	CD8 T cells	1. Plasma TNF α, IFN‐γ, IL‐33 levels increased in the APOE ε4 carriers but IL‐7 expression notably decreased. 2.A negative correlation was observed between plasma IL‐7 level and the hippocampal atrophy degree. 3. the expression of IL‐7R and CD28 also decreased in PBMCs of APOE ε4 carriers. 4. ScRNA‐seq data results indicated that the changes were mainly related to the CD4+ Tem (effector memory) and CD8+ Tem T cells.	The First Affiliated Hospital of Shantou University Medical College, Shantou, China; The Second Affiliated Hospital of Shantou University Medical College, Shantou, China; The Second Hospital of Shandong University, Jinan, China; Fudan University Huashan Hospital, Shanghai Medical College Fudan University, Shanghai, China; Shantou University Medical College, Shantou, China
	[111]		P301S − / − , P301S + /−, and P301S + /+ mice	GZMK+CD8+T cells	1. Using mice that express mutant human tau in neurons, it was shown that microglia slowed tauopathy development by controlling the spread of pTau in the central nervous system and blood. However, over time microglia converted into distressed antigen‐presenting cells, acquired neuronal transcripts and were targeted by resident, clonally expanded CD8 + T cells. 2. These cells did not express traditional effector molecules, such as IFNγ, TNF or granzymes a/b/c, but instead deposited granzyme K (GZMK) onto microglia and were regulated by immune checkpoint proteins (TIGIT, PD‐1), as blockade of TIGIT and PD‐1 enhanced disease progression. GZMK + CD8 + T cells also targeted microglia in pTau‐rich human brain lesions resulting from age, Alzheimer's disease or chronic traumatic encephalopathy. 3. Deletion of CD8 + T cells in mice promoted the emergence of distressed microglia containing neuronal transcripts, markedly enhanced pTau spread and accelerated neurological decline.	National Institute of Neurological Disorders and Stroke (NINDS), National Institutes of Health (NIH), Bethesda, MD, USA; National Institute of Allergy & Infectious Diseases, NIH, Bethesda, MD, USA; National Institute of Neurological Disorders and Stroke, NIH, Bethesda, MD, USA; Uniformed Services University of the Health Sciences, Bethesda, MD, USA; School of Nursing, Johns Hopkins University, Baltimore, MD, USA

*Note:* CD8 + T effector memory CD45RA+ (TEMRA) cells, Alzheimer's disease (AD), cerebrospinal fluid (CSF), T cell receptor (TCR), major histocompatibility complex (MHC), peripheral blood mononuclear cells (PBMCs), mild cognitive impairment (MCI), amyloid‐β (Aβ), CD3 + CD8 + CD27 − T effector memory CD45RA+ (TEMRA), T central memory (TCM), T effector memory (TEM) cells, Alzheimer's disease (AD), Epstein‐Barr virus (EBV), interferon‐γ (IFN = γ), the C‐X‐C motif chemokine ligand 10 (CXCL10), C‐X‐C motif chemokine receptor 3 (CXCR3), ATP6V1D: ATPase H+ transporting V1 subunit D, risk gene orthologs vha‐10 (ATP6V1G2), carrot‐made LTB‐Syn antigen (cLTB‐Syn), synaptic vesicle cycling (SVC), SVC related genes (SVCRGs), Immunity‐related genes (IRGs), single‐cell RNA(scRNA), CD8+ resident memory T cells (TRM), protease‐activated receptor‐1 (PAR‐1), granzyme K (GrK), bulk RNA sequencing (RNA‐seq), single‐cell RNA sequencing (scRNA‐seq), tumor necrosis factor α(TNF α), interferon γ(IFN‐γ), interleukin (IL), apolipoprotein E (APOE), mild cognitive impairment (MCI), C‐X‐C Motif Chemokine Receptor 6 (CXCR6), C‐X‐C Motif Chemokine Ligand (CXCL16), phosphorylated tau (pTau), granzyme K (GZMK), phosphorylated tau (pTau).

### 4.3 The Role of Anti‐Aβ‐Specific CD8+ T Cells Versus CD4+ T Cells in AD

In 2023, Afsar et al. compared the role of anti‐Aβ‐specific CD8+ T cells versus CD4+ T cells in AD. They suggested that (1) in healthy controls, 90% of infiltrated cells are T cells, with a CD4:CD8 ratio of 3.5 [112]. However, the CD4/CD8 ratio was inverted below 1.00 (about 8% in the age range of 20–59 years and around 16% in the age range of 60–94 years [113]. A similar study [114] showed that, compared with CD8+ T cells in healthy controls, there were large changes in CD4+ in lower proportions of naïve cells, more late‐differentiated cells, and higher percentages of activated CD4+ CD25+ T cells without a Treg phenotype in AD patients [47]. It was shown that, compared with the healthy controls, patients with AD had a higher CD4+∕CD8+ ratio and a lower percentage of CD8+ T cells [115]. Moreover, an earlier report showed that the increasing CD4+∕CD8+ ratio was due to a decrease in CD8+ T cells in patients with AD [116]. Also, the percentage of CD4+ T cells increased in patients with AD [117]. Furthermore, T cell subsets had distinct patterns in different subtypes of patients with AD. For example, CD4+ T cells were increased in patients with mild or moderately severe AD [50, 118], whereas CD8+ T cells were decreased in those with severe AD [119]. Specifically, the increased apoptosis in peripheral lymphocytes of the patients was largely caused by CD4+ cells rather than CD8+ T cells [120]. CD8+ T cells were increased in healthy controls; however, they were increased in patients with AD [121].

## 5 Future Prospects

Many subsets of CD8+ T cells, such as Tc1, Tc2, Tc17, and CD8+ Tregs, have been well‐studied in autoimmunity [65, 122, 123, 124]. These subsets may be of interest in future AD research, given that CD8+ T cells are not cytotoxic T cells. The protocol used for evaluating CD4+ T cells in AD studies can also be followed to elucidate the roles of CD8+ T cells in this disease.

In fact, many reports on AD have focused more on the role of CD4+ T cells rather than on CD8+ T cells; however, the reason is still unclear. Some studies compared the roles of CD4+ versus CD8+ T cells depending on the severity of AD, as discussed in Section 4.3. Unfortunately, there is an ongoing debate [125, 126], for example, regarding the notable decrease in both CD4+ and CD8+ T cells in patients with AD [127]. Therefore, the actual role of CD8+ T cells in patients with AD remains to be fully elucidated, which is our goal for future studies.

## Author Contributions

Yong Peng received funding support and developed the research hypothesis. Yong Peng, Shun‐yu Yao, Si‐Liang Wu, Huan Yang, Xiuli Zhang, Sugimoto Kazuo, Jia Liu, Miao‐qiao Du, Lan‐xin Lin, and Xu‐hui Kang drafted the primary manuscript, which was the result of collaborative writing efforts by all authors.

## Conflicts of Interest

The authors assert that the study was carried out in the absence of any commercial or financial ties that might be perceived as potential conflicts of interest.

## AI Usage Declaration

The authors assert that this manuscript did not use AI in preparing the main text and figures.

## Data Availability

The availability of our data is in our submission.
